# Mutation of the *RDR1* gene caused genome-wide changes in gene expression, regional variation in small RNA clusters and localized alteration in DNA methylation in rice

**DOI:** 10.1186/1471-2229-14-177

**Published:** 2014-06-30

**Authors:** Ningning Wang, Di Zhang, Zhenhui Wang, Hongwei Xun, Jian Ma, Hui Wang, Wei Huang, Ying Liu, Xiuyun Lin, Ning Li, Xiufang Ou, Chunyu Zhang, Ming-Bo Wang, Bao Liu

**Affiliations:** 1 Key Laboratory of Molecular Epigenetics of Ministry of Education (MOE), Northeast Normal University, Changchun 130024, China; 2 Faculty of Agronomy, Jilin Agricultural University, Changchun 130118, China; 3 Jilin Academy of Agricultural Sciences, Changchun 130033, China; 4 School of Food Production Technology and Biotechnology, Changchun Vocational Institute of Technology, Changchun, China; 5 Commonwealth Scientific and Industrial Research Organisation Plant Industry, Canberra, Australian Capital Territory 2601, Australia

**Keywords:** Gene expression, Epigenetics, Small RNA, DNA methylation, *RDR1*, *Oryza sativa* L

## Abstract

**Background:**

Endogenous small (sm) RNAs (primarily si- and miRNAs) are important *trans/cis-*acting regulators involved in diverse cellular functions. In plants, the RNA-dependent RNA polymerases (RDRs) are essential for smRNA biogenesis. It has been established that RDR2 is involved in the 24 nt siRNA-dependent RNA-directed DNA methylation (RdDM) pathway. Recent studies have suggested that RDR1 is involved in a second RdDM pathway that relies mostly on 21 nt smRNAs and functions to silence a subset of genomic loci that are usually refractory to the normal RdDM pathway in *Arabidopsis*. Whether and to what extent the homologs of *RDR1* may have similar functions in other plants remained unknown.

**Results:**

We characterized a loss-of-function mutant (*Osrdr1*) of the *OsRDR1* gene in rice (*Oryza sativa* L.) derived from a retrotransposon *Tos17* insertion. Microarray analysis identified 1,175 differentially expressed genes (5.2% of all expressed genes in the shoot-tip tissue of rice) between *Osrdr1* and WT, of which 896 and 279 genes were up- and down-regulated, respectively, in *Osrdr1*. smRNA sequencing revealed regional alterations in smRNA clusters across the rice genome. Some of the regions with altered smRNA clusters were associated with changes in DNA methylation. In addition, altered expression of several miRNAs was detected in *Osrdr1*, and at least some of which were associated with altered expression of predicted miRNA target genes. Despite these changes, no phenotypic difference was identified in *Osrdr1* relative to WT under normal condition; however, ephemeral phenotypic fluctuations occurred under some abiotic stress conditions.

**Conclusions:**

Our results showed that *OsRDR1* plays a role in regulating a substantial number of endogenous genes with diverse functions in rice through smRNA-mediated pathways involving DNA methylation, and which participates in abiotic stress response.

## Background

RNA silencing is an evolutionally conserved gene regulation mechanism in eukaryotes mediated by 20–25 nt non-coding small (sm)RNAs. These smRNAs are processed from double-stranded (ds) or hairpin RNA molecules by Dicer-like (DCL) proteins, and guide RNA-induced silencing complexes to cognate single-stranded RNAs based on sequence complementarity, and result in degradation of the targeted RNAs [1-3]. In plants, there are several different classes of smRNAs, including 20–24 nt micro RNAs (miRNAs) processed by DCL1, 21–22 nt small interfering RNAs (siRNAs) by DCL4 and DCL2, and the 24 nt heterochromatin-associated siRNAs by DCL3. miRNAs play an important role in plant development by directing posttranscriptional gene silencing (PTGS) of regulatory genes such as those encoding transcription factors. Similarly, 21–22 nt siRNAs guide the degradation of viral RNAs as well as some endogenous mRNAs and are important for plant defense against viruses and for some aspects of plant development [4-6]. Unlike these PTGS-associated smRNAs, the 24 nt siRNAs are associated with RNA-directed DNA methylation (RdDM), a plant-specific *de novo* DNA methylation pathway required for transcriptional silencing of transposable elements and other DNA repeats to maintain genome stability [7-10].

The biogenesis of siRNAs in plants requires the activity of RNA-dependent RNA polymerase (RDR), which converts single-stranded RNAs to dsRNA precursors of siRNAs. The dicot model plant *Arabidopsis thaliana* has six *RDR* genes, i.e., *RDR1*, *RDR2*, *RDR3a*, *RDR3b*, *RDR3c* and *RDR6*[11], of which three RDRs (*RDR1*, *RDR2* and *RDR6*) are shown to play roles in the RNA silencing pathways. *RDR2* is required for 24 nt siRNA biogenesis and therefore involved in the canonical RdDM pathway [7-9]. *RDR6* is involved in the production of the endogenous 21 nt *trans*-acting siRNAs and also essential for sense transgene-induced PTGS [12,13]. Both *RDR6* and *RDR2* are also involved in viral siRNA accumulation in infected *Arabidopsis plants*[14-16]. The function of *RDR1* in RNA silencing is less understood, but recent studies have shown that it is involved in siRNA biogenesis from a subset of RNA viruses [17-19]. Furthermore, *rdr1* mutant of *Arabidopsis* showed loss of DNA methylation in a subset of genomic loci in comparison to wild-type *Arabidopsis* plants [20,21], suggesting that *RDR1* plays a role in the recently identified non-canonical, 21 nt siRNA-directed RdDM pathway [20,21]. However, the function of *RDR1* in gene regulation from a genome-wide perspective has not been investigated in any plant.

In contrast to *Arabidopsis* that has six *RDR* genes, the RDR family of rice (*Oryza sativa* L.), a model plant for monocots, contains only three members, namely *OsRDR1*, *OsRDR2* and *OsRDR6*[11,22,23]. A previous study showed that *OsRDR1* has a similar function to its counterparts in *Arabidopsis* and tobacco (*Nicotiana tabacum*) in PTGS-based silencing of certain RNA viruses, such as Bromovirus [6,19,24]. To investigate if *OsRDR1* plays a role in regulation of endogenous genes in rice, we characterized a loss-of-function mutant of *OsRDR1* derived from a disruptive LTR retrotransposon (*Tos17*) insertion into the 2nd exon of the gene. We investigated genome-wide changes in gene expression and smRNA profiles, localized changes in DNA methylation, and phenotypes under normal and several abiotic stress conditions in this rice *rdr1* mutant.

## Results

### Characterization of the *rice RDR1* mutant (*Osrdr1*)

**Figure 1:**
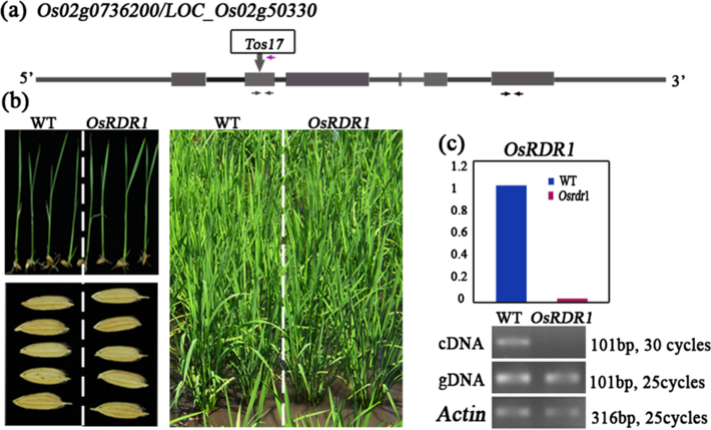
***OsRDR1 *gene expression is abolished in the *Tos17 *insertion mutant *Osrdr1*. (a)** Structure of the *OsRDR1* locus with the *Tos17* insertion into the 2nd exon (vertical arrows). Heterozygous/homozygous individuals were selected based on PCR which primers were indicated by the purple arrows while primers for *OsRDR1* expression analysis were indicated by black arrows. **(b)** Germinated seedling, paddy-field-grown plant and kernel phenotypes of wide-type (WT) and *Osrdr1*. **(c)** Relative to WT, *OsRDR1* expression was silenced in shoots of *Osrdr1*, as evidenced by both qRT-PCR (top) and semi-quantitative RT-PCR (bottom) amplifications with gene-specific primers downstream of the *Tos17* insertion (horizontal arrows). Genomic DNAs were used as positive controls.

We obtained a LTR retrotransposon *Tos17*[25] insertion line for *OsRDR1* (accession number H0643) from the *Tos17* insertion mutant library of rice cv. *Hitomebore* (http://www.cns.fr/spip/Oryza-sativa-retrotransposon-Tos17.html). Molecular characterization identified H0643 as heterozygous for a *Tos17* insertion into the second exon of *OsRDR1* (Figure 1a). We obtained the homozygous mutant (*OsRDR1*−/− or *Osrdr1*) and its sibling wild type (WT) plants by selfing of the heterozygous plant (*OsRDR1*+/−) for five successive generations. In each generation, the three kinds of genotypes, WT, heterozygote and homozygous mutant, were selected based on locus-specific PCR amplifications (Figure 1a). Both the heterozygous and homozygous plants for *OsRDR1* showed no discernibly altered phenotypes in the entire growth and developmental period over multiple generations under normal field conditions (Figure 1b). Semi-quantitative and real-time quantitative (q)RT-PCR analyses confirmed that the homozygous *OsRDR1* mutant (*Osrdr1*) had a complete loss of *OsRDR1* expression in shoot-tip tissue wherein the gene was highly expressed in WT plants (Figure 1c). This indicated that the exonic insertion of *Tos17* knocked out the expression of *OsRDR1*, and hence, abolished its function.

### Genome-wide changes in gene expression in *Osrdr1*

**Figure 2:**
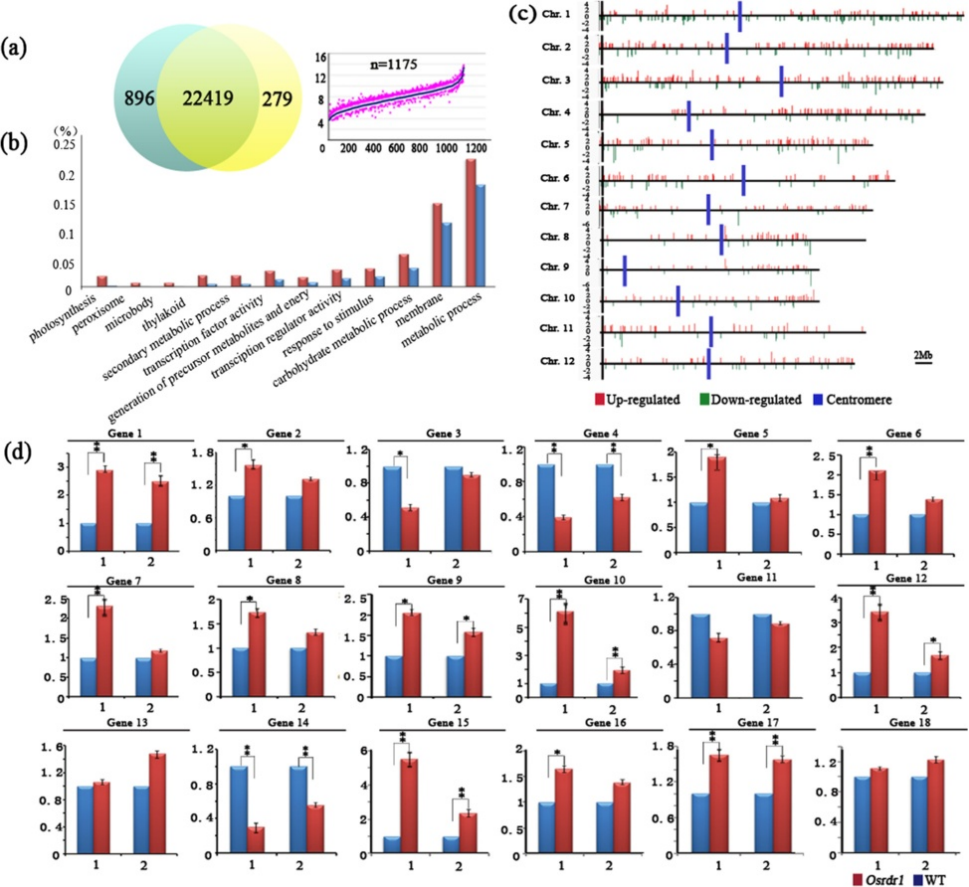
**Effects of null mutation of *OsRDR1 *on global gene expression in rice. (a)** A summary of microarray analysis showing the total number of genes detected and the number of differentially expressed genes in WT and *Osrdr1*. **(b)** Gene Ontology (GO) category analysis of the 1,175 differentially expressed genes between WT and *Osrdr1* . The *y*-axis is the percentage of genes mapped by the GO category terms: the percentages were calculated by the number of *Osrdr1* vs. WT differentially expressed genes divided by the total number of genes mapped to the particular GO category. The *x*-axis is the GO category terms which were ordered by their relative abundance (total number of expressed genes in the shoot-tip tissue of rice are 45,078). The blue bars denote percentages for each category of all the annotated genes, and the red bars denote percentages of the GO categories of all the expressed genes. **(c)** Distribution of the 1,175 *Osrdr1* vs. WT differentially expressed genes across each of the 12 the rice chromosomes (horizontal lines). The red columns above and the green columns below the chromosomes represent up- and down-regulated genes in *Osrdr1* vs. WT, respectively. The *y*-axis indicates fold changes in gene expression between *Osrdr1* and WT. The vertical blue bars denote for centromeric regions. **(d) **Validation of the microarray results by qRT-PCR, where the blue and red columns represent WT and *Osrdr1*, respectively. Numbers 1 and 2 below each gene represents data of microarray and qRT-PCR results, respectively. Statistical significance at that *P* <0.05 and *P* <0.01 levels is marked by one or two asterisks.

We profiled the transcriptome of shoot-tip tissues between *Osrdr1* and its sibling WT plants using the Affymetrix GeneChip Rice® Genome Array (The Affymetrix, Inc. Santa Clara, CA, USA). After normalization of the microarray data, we detected 57,381 expressed genes in the shoot-tip tissue of rice. The expression levels of 22,419 genes were conserved between *Osrdr1* and WT, but 896 and 279 genes showed significant up- and down-regulation in *Osrdr1*, respectively (Figure 2a). A Gene Ontology (GO) category analysis of these 1,175 differentially expressed genes showed that they were enriched in a variety of GO categories (Figure 2b). However, these 1,175 differentially expressed genes were found to distribute non-randomly across the 12 rice chromosomes (P = 2.2E-16, based on Chi square test). For example, chromosomes 1, 2 and 3 contained significantly more distributions than the rest chromosomes (Figure 2c). It is also clear from the data that within a given chromosome, the distribution is also nonrandom, for example, the distributions are almost exclusively confined to the long arms of chromosomes 4 and 8 and 9 relative to their respective short-arms (Figure 2c).

The highly reproducible microarray profiles among three biological replicates for both *Osrdr1* and its WT sibling plants testified reliability of the data and their analysis. All microarray data have been submitted to the GEO repository under the accession number of GSE58007. To further verify the quality of the microarray data and analysis, we analyzed 18 genes representing both up- and down-regulation in *Osrdr1 vs.* WT, as well as equal expression between the two lines using qRT-PCR assay on the same cDNAs as used for microarray. The qRT-PCR results were highly consistent with the microarray data for almost all the 18 tested genes in levels or at least in trends of expression changes (Figure 2d), confirming reliability of the microarray analysis.

### Alteration in smRNA clusters in *Osrdr1*

**Figure 3:**
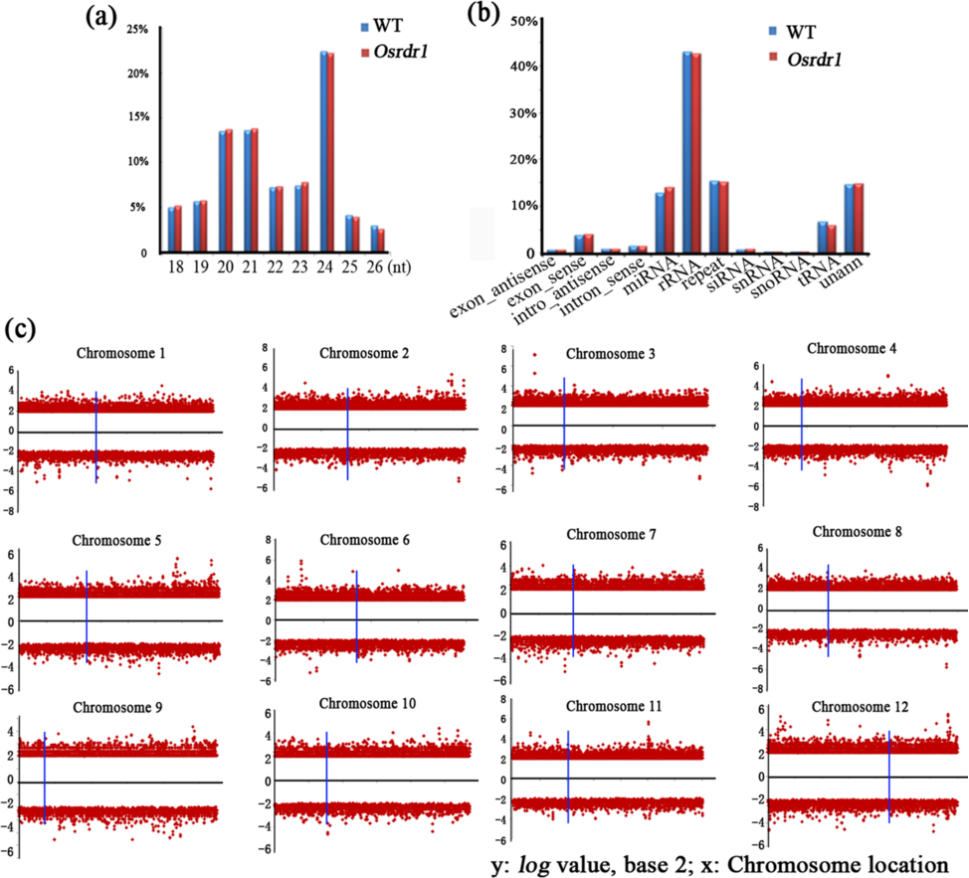
**Effects of null mutation of *OsRDR1 *on genome-wide profiles of smRNAs in rice. (a)** Size distribution of smRNA from *Osrdr1* and WT; **(b)** Categories of smRNAs from *Osrdr1* and WT; the red and blue columns represent *Osrdr1* and WT, respectively. **(c)** Difference of smRNA clusters (RPM) within 100 bp sliding windows across each of the 12 rice chromosomes between *Osrdr1* and WT, where *x* axis is the length of chromosome and *y* axis is the value of different RPMs (log value, base 2). The vertical blue lines denote centromeric regions in each chromosome.

Previous studies have established that *RDR1* function is required for biogenesis and/or amplification of some types of RNA virus-related smRNA accumulation in *Arabidopsis*[26] and tobacco (*Nicotiana tabacum*) [27]. These findings promoted us to test whether loss of function of *OsRDR1* may have a general impact on “normal” smRNA abundance in rice, and we investigated this issue by high-throughput smRNA sequencing. Comparison of the 10,398,592 clean smRNA reads from *Osrdr1* with the 9,339,435 reads from its sibling WT (*see* Methods) revealed highly similar profiles in both size distributions and sequence categories of the smRNAs between *Osrdr1* and WT (Figure 3a, b), suggesting that the overall smRNA abundance was not generally affected by the loss of function of *OsRDR1*.

Genome-wide overall similarity in smRNA abundance does not necessitates absence of smRNA fluctuations in localized smRNA clusters, because up- and down-regulated smRNA accumulation can be masked by reciprocal compensation. We thus investigated localized smRNA accumulation between *Osrdr1* and its sibling WT by mapping the cleaned smRNA reads to 100 bp sliding windows (being reflected as smRNA clusters) across each of the 12 rice chromosomes, normalized the reads to Reads per Million (RPM), and then compared the RPM smRNA clusters between *Osrdr1* and WT. Using 4-fold difference as a cut-off threshold, we identified many smRNA clusters with differential abundance between *Osrdr1* and its sibling WT, which were uniformly distributed across the entire length of each chromosome (Figure 3c). Next, we extracted the differentially expressed smRNAs between *Osrdr1* and WT (also based on a cut-off threshold of 4-fold difference) in the size range of 20-24 nt, which should parsimoniously contain all siRNAs, and mapped them to the same 100 bp windows across each chromosome. We found that this subset of differential smRNA clusters also distribute on both arms of each chromosome (Additional file 1), although due to their smaller numbers, we cannot rule out the possibility that the distribution might show “hot spots” within a given chromosome. Taken together, the smRNA sequencing data suggested that loss-of-function mutation in *OsRDR1* caused extensive alterations in smRNA clusters across each chromosome and throughout the genome, but it did not result in marked fluctuations of overall smRNA profiles, probably due to more or less equally increased and decreased abundance of the smRNA clusters which offset each other.

### Altered expression of miRNAs and their target genes in *Osrdr1*

**Figure 4:**
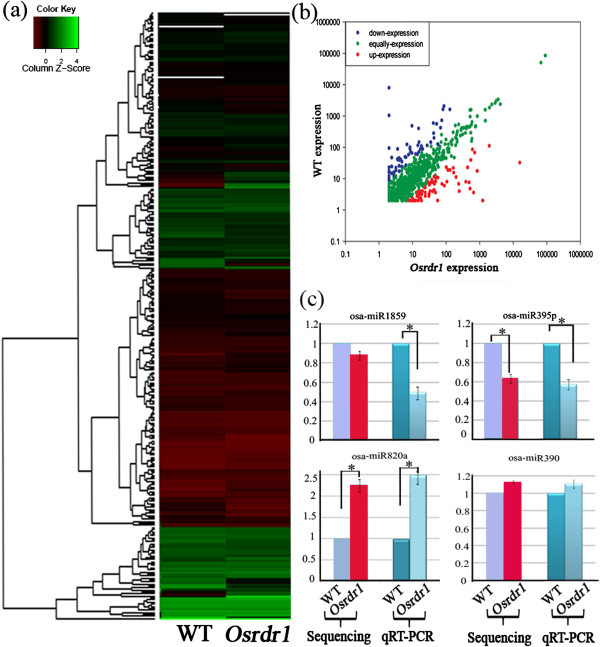
**Effects of null mutation of *OsRDR1 *on abundances of miRNAs in rice. (a)** Expression comparison of known miRNAs between *Osrdr1* and WT. **(b)** Three patterns of expression changes of known miRNAs. The red and blue spots represent up and down-regulated in *Osrdr1*, respectively; the green spots represent no variation between *Osrdr1* and WT. **(c)** qRT-PCR to verify variation of miRNA accumulation between *Osrdr1* and WT. Statistical significance is marked by asterisks.

Given the diverse important roles played by miRNAs, we investigated if their accumulation might be affected in the *Osrdr1* mutant*.* Previous computational and cloning studies have identified *ca.* 300 miRNAs from 86 miRNA families in rice [28,29]. Based on this information, we first analyzed the abundance of known rice miRNAs (Osa-miRNAs) (listed in miRBase14.0) in *Osrdr1* and WT. This analysis (Figure 4a, b; Additional file 2) indicated that: (1) majority (90.7%) of the known Osa-miRNAs were expressed equally or nearly so between *Osrdr1* and WT; (2) some miRNAs (5%) showed > 2 fold increased expression in *Osrdr1* relative to WT, with the highest expression ratio reaching 9.0:1 (*t*-test, P < 0.05); (3) some miRNAs (4.3%) showed >2 fold decrease in expression in *Osrdr1* relative to WT, with the lowest expression ratio of 1:6.1 observed for miR167j between mutant and WT (*t*-test, P < 0.05); (4) Osa-miR395p and Osa-miR395s, being from the same miRNA family, showed changes in expression to opposite directions, with an expression ratio of 1:5.6 for osa-miR395p but 4.2:1 for osa-miR395s in mutant vs. WT (*t*-test, P < 0.05). To verify the expression differences based on the smRNA sequencing data, we performed semi-nested qRT-PCR analysis of four miRNAs in mutant and WT. The qRT-PCR results were found consistent with the smRNA sequencing data (Figure 4c), confirming the changes in miRNA expression between *Osrdr1* and WT.

In addition to the known miRNAs, we identified a total of 10 putative novel osa-miRNAs from the smRNA data of the mutant and WT plants based on prediction of pre-miRNA-like stem-loop structures in sequences surrounding the smRNA sequences in the rice genome (Additional file 3). Three of these novel miRNAs (Osr-miRNA-N5.1, −N5.2 and -N5.3) had an identical mature sequence but corresponded to three independent genomic loci, thereby forming a novel miRNA family. For the putative miRNA Osr-miRNA-N7, smRNA reads were detected from both the 5’ (5p) and the 3’ (3p) half of the predicted stem-loop structure, but the 5p smRNA showed a higher abundance than the 3p smRNA (Additional file 3), indicating that the 5p smRNA is the guide strand (miRNA) whereas the 3p smRNA is the passenger strand (miRNA*) [30]. Like the known miRNAs, these novel miRNAs also showed expression variation between *Osrdr1* and WT, with 3 showing expression only in *Osrdr1*, and 5 showing expression only in WT plants (Additional file 3). Taken together, our results suggest that *OsRDR1* was likely involved in miRNA accumulation in rice.

To investigate if the altered miRNA accumulation in *Osrdr1* relative to WT was associated with changes in miRNA target gene expression, we compared the miRNA expression profiles (abundance) derived from the smRNA sequencing data with the target gene expression levels based on the microarray data. We did not find a generalized relationship between the miRNA abundance and target gene expression levels (Additional file 4a). Instead, four types of relationships were recognized for a subset of miRNAs and their predicted targets (Additional file 4b), which included: (1) reduced miRNA abundance was correlated with up-regulated expression of target genes in *Osrdr1* relative to WT (Additional file 4b-i); (2) increased miRNA abundance was correlated with down-regulated expression of target genes in *Osrdr1* relative to WT (Additional file 4b-ii); (3) both miRNAs and their target genes were up-regulated in *Osrdr1* relative to WT (Additional file 4b-iii); (4) both miRNAs and their target genes were down-regulated in *Osrdr1* relative to WT (Additional file 4b-iv). The first two types of relationships supported a role of miRNAs in down-regulating expression of their predicted target genes. The last two types of relationships could be a result of concordant transcriptional regulation of the miRNAs and their target genes caused by another more upstream regulator(s) whose expression or activity was modified due to loss of function of *OsRDR1.* All small RNA data have been submitted to GenBank under the accession numbers of SRP042238.

### Locus-specific alteration of DNA methylation in *Osrdr1*

As the *Arabidopsis RDR1* has been shown to play a role in the non-canonical, NERD-dependent RdDM pathway [20,21], we were interested to know if *OsRDR1* might have a similar function in rice. We therefore examined cytosine methylation and gene expression levels of 10 selected genomic loci in *Osrdr1* and its sibling WT using bisulphite sequencing and qRT-PCR analysis. These loci overlapped with two transposable elements (TEs) and three protein-coding genes, which were chosen as representatives because they all showed alteration in smRNA clusters in *Osrdr1* relative to WT (Additional file 5). The bisulphite-sequenced regions for the two TEs (retrotransposon *Tos17* and DNA transposon *Pong*) included: (1) portions of the 5’- and 3’-LTRs together with their immediate flanking regions of two *Tos17* copies (located on chromosomes 10 and 7, respectively) (Additional file 5a, b); (2) the 5’ termini along with their immediate flanking regions of two *Pong* copies (located on chromosomes 2 and 9, respectively) (Additional file 5c, d), and; (3) a body-region of the transposase-encoding ORF of *Pong* (Additional file 5e) that is shared by all conserved copies of the element. The bisulphite-sequenced regions of the three protein-coding genes are all within their 5’-upstream regions (Additional file 5f, g, h).

**Figure 5:**
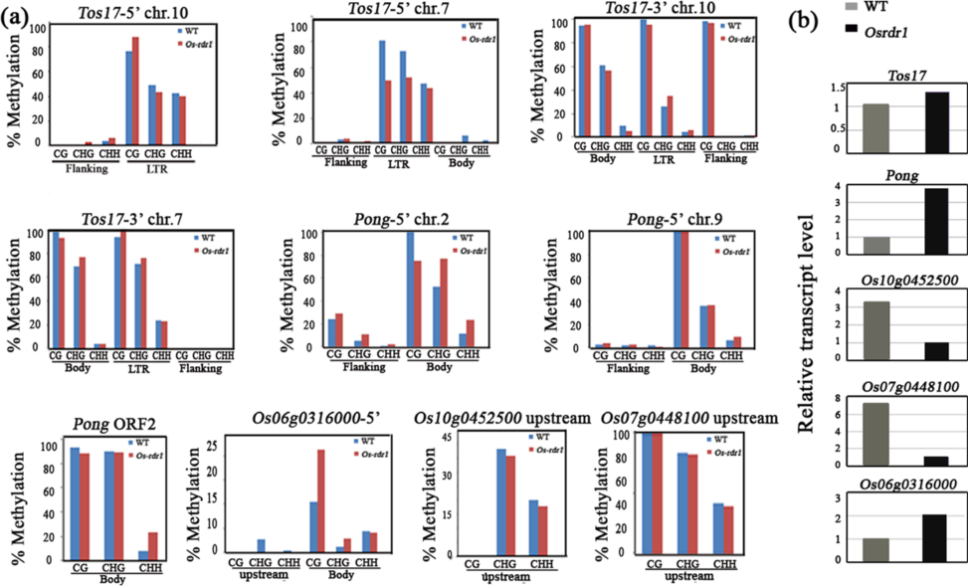
**Effects of null mutation of *OsRDR1 *on locus-specific alterations of DNA methylation in rice. (a)** Alteration in DNA methylation between *Osrdr1* and WT in the three cytosine sequence contexts, CG, CHG and CHH, based on bisulphite sequencing of 10 genomic loci from two transposable elements (TEs), *Tos17* (four regions) and *Pong* (three regions) and three genes (one region each). **(b)** Expression differences of the TEs and genes between *Osrdr1* and WT based on qRT-PCR analysis.

The bisulphite sequencing results showed that: (1) of the five *Tos17* regions analyzed, only the 5’ LTR region for the *Tos17* copy located on chromosome 7 showed marked decrease (by *ca.* 30%) in CG and CHG methylation but not in CHH methylation in *Osrdr1* relative to WT (Figure 5a and Additional file 5b); (2) for the three *Pong* regions analyzed, only the 5’region of the copy located on chromosome 2 showed clear methylation changes: decrease in CG methylation by 20% and increase in both CHG and CHH methylation by approximately 30% and 50%, respectively, in *Osrdr1* relative to WT (Figure 5a and Additional file 5c); of the three genic loci analyzed, only one (*Os*06g0316000) showed increase in CG methylation by *ca.* 50% in *Osrdr1* relative to WT, while methylation of the other two regions were unchanged (Figure 5a and Additional file 5f).

The two TEs showed significant up-regulation in *Osrdr1* relative to WT (Figure 5b), consistent with a decrease in methylation at the 5’ regions of one copy of each TEs in *Osrdr1* (Figure 5a). Notably, all the three genes analyzed did not show the expected relationship between DNA methylation state of their 5’-regulatory regions and expression levels. Specifically, one gene (*Os*06g0316000) that showed increase in CG methylation in *Osrdr1* was up-regulated in expression (Figure 5b); the remaining two genes showed down-regulation in *Osrdr1* relative to WT despite the lack of methylation changes in the bisulphite-sequenced regions (Figure 5b).

We next investigated possible relationships between smRNA accumulation and DNA methylation. We found that almost all of the altered CHH methylation was associated with changes in smRNA clusters. For example, the increased CHH methylation of the *Pong* copy located on chromosome 2 was associated with a moderate increase in smRNA accumulation, whereas the slight decrease in CHH methylation in the flanking region and the increase in CHH methylation in the gene body region of the *Pong* copy located on chromosome 9 were associated with moderate decrease and increase in smRNA accumulations, respectively (Additional file 5c). These positive correlations of smRNA accumulation and CHH methylation suggests that *OsRDR1* plays a role in the *de novo* CHH methylation in a subset of genomic loci in rice, probably by affecting the production/accumulation of smRNAs required for RdDM, as shown in *Arabidopsis*[20,21]. The locus-specificity of methylation changes or the two analyzed TEs indicated that their methylation patterns were determined by either or both the flanking sequences and the local chromatin environment, an issue which warrants further investigations.

### Phenotypes in *Osrdr1* under normal and abiotic stress conditions

**Figure 6:**
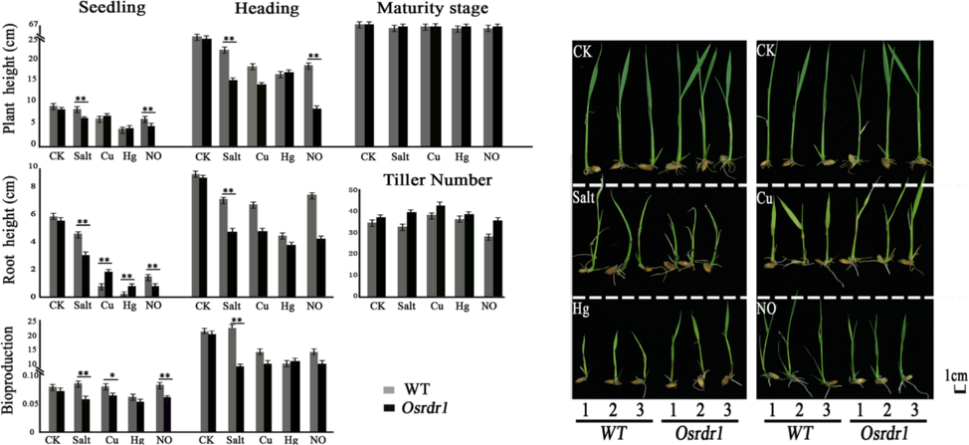
**Effects of null mutation of *OsRDR1 *on phenotypes under normal and abiotic stress conditions in rice.** Phenotyping of *Osrdr1* and WT plants at three growth stages under normal condition and four abiotic stress conditions (salt, heavy metal Cu^2+^, heavy metal Hg^2+^ and overdose NO).

It is known that various stress conditions may produce protracted effects on genome stability, leading to transgenerational changes in genome structure, which are proposed to have been initiated by epigenetic mechanisms [31-37]. We were therefore interested to know if *OsRDR1* may play a role in stress response in rice. We quantified phenotypes between *Osrdr1* and WT plants under normal and several short-term abiotic stress conditions (*see* Materials and Methods), which included treatments with salt, heavy metals Cu^2+^and Hg^2+^, and overdose nitric oxide (NO). The results showed that no phenotypic difference was found between *Osrdr1* and WT under normal condition, but significant ephemeral phenotypic differences between the two genotypes emerged in some of the different stress conditions (Figure 6). Specifically, (1) seedlings of *O*s*rdr1* were more sensitive than WT to salt and overdose NO treatments, as being reflected by reduced plant height, root length and biomass at the seedling stage, with the difference in plant height and root length being persisted to the heading stage after removal of the stresses; (2) Seedlings of *Osrdr1* showed increased tolerance to heavy metal Cu^2+^/Hg^2+^ as indicated by increased root length, but upon removal of the stresses the differences were gradually attenuated and completely disappeared at the heading stage; (3) When both unstressed and the transiently-stressed plants of the mutant and WT were grown to maturity, no difference in plant height, tiller number, panicle and kernel traits was observed between the two genotypes. Collectively, our results suggest that *OsRDR1* has a potential function in stress response in rice, but the effects are contingent with presence of stresses without exerting protracted influence when the stresses are removed.

## Discussion

RNA silencing pathways have been well characterized in the dicot model plant *Arabidopsis* but remain poorly studied in other plants like monocots to which many major crops belong. Utilizing a retransposon *Tos17* insertion mutant of *OsRDR1* in rice, we performed genome-wide analysis to unveil the function of *OsRDR1*, a component recently shown in *Arabidopsis* to be involved in non-canonical, 21 nt siRNA-directed RdDM pathway [20,21], on expression of endogenous genes. By deep sequencing of smRNAs and microarray analysis of gene expression in the *Osrdr1* mutant and its sibling WT, we showed that the expression of > 1,000 genes were significantly changed in *Osrdr1* relative to WT, suggesting that *OsRDR1* plays a role in genome-wide gene regulation in rice. In addition, the *Osrdr1* mutant showed regional alterations in smRNA accumulation and/or titration across the rice genome, and at least some of which are associated with locus-specific alteration of DNA methylation.

Among the differentially accumulated smRNAs, many were miRNAs both previously known and newly identified in this study. Expression changes in many of these miRNAs are associated with changes in their target gene expression, which could partly be responsible for the gene expression changes observed in the *Osrdr1* mutant relative to WT. The mechanism by which mutation of *OsRDR1* caused changes in miRNA expression in rice was not clear. It was found in *Arabidopsis* that none of the RDRs has a direct role in miRNA biogenesis [2,38]. However, RDRs could impact miRNA accumulation indirectly by either affecting miRNA precursor gene expression through the TGS or PTGS pathways [1,2,38] or generating dsRNAs that compete for DCL1 function that is required for miRNA biogenesis [1,2].

Our results suggested that *OsRDR1* might play a role in maintaining the intrinsic locus-specific DNA methylation patterns, as its mutation caused alteration of methylation at some of the loci we analyzed. In particular, the changes in CHH methylation, which are indicative of *de novo* methylation by the RdDM pathway, showed correlation with changes in smRNA accumulation. In the respect of reduced CHH methylation and concomitant reduced smRNA accumulation, *OsRDR1* may functionally resemble the *Arabidopsis RDR1* and plays a role in the 21 nt siRNA-dependent non-canonical RdDM pathway [20,21]. However, some of the analyzed loci showed increased CHH methylation that is associated with increased smRNA accumulation in *Osrdr1*. We should caution that because we analyzed only 10 loci, the results observed may not be extrapolated to global scale. In *Arabidopsis*, it was documented that mutation of *RDR1* resulted in near complete loss of methylated cytosine of all three sequence contexts (CG, CHG and CHH) within the 4,949 CHH hypomethylation DMRs (differentially methylated regions) between *drm*1/2 and WT [20]. Therefore, genome-wide methylation analysis (methylome) of the *Osrdr1* mutant will be required to confirm whether *OsRDR1* plays a similar role globally in rice.

Previous studies in *Arabidopsis* and *Nicotiana* have defined an established role of RDR1 in plant virus responses [24,26,27]. We showed here that *Osrdr1* exhibited no phenotypic differences from its sibling WT plants under normal growing condition, but displayed ephemeral phenotypic fluctuations contingent with presence of several abiotic stress conditions. This observation, together with the enriched GO categories including those involved in metabolic process of the differentially expressed genes between *Osrdr1* and WT (Figure 2b), suggest that the effects of altered gene expression due to *OsRDR1* mutation has been largely canalized under normal condition but can be released by certain abiotic stress conditions [39], an issue that merits further investigations. Regardless, our results suggest that, apart from its established role in the production and amplification of exogenous, virus-derived siRNAs (vsiRNAs) in infected plants [26,27], the rice *RDR1* homolog (*OsRDR1*) might also play a role in certain abiotic stress responses, which however may not involve stable epigenetic changes in this respect. In this regard, it should be emphasized again that the genome-wide analyses of both smRNA profiles and gene expression in *Osrdr1* were conducted on plants grown under normal conditions. Therefore, further studies are needed to conduct the analyses in plants of the mutant and WT under both short- and long-term stress conditions. It would also be interesting to analyze the progeny of stress-treated *Osrdr1* plants to investigate if *OsRDR1* is involved in transgenerational inheritance of stress-induced epigenetic changes, if they occurred.

## Conclusions

How *RDR1* affects global gene expression and smRNA profiles have not been previously investigated in any plant species. By analyzing a null mutant of the rice *RDR1* gene (*OsRDR1*), we showed that expression of more than 1,000 endogenous genes of diverse gene ontology (GO) categories were significantly altered in the mutant, indicating a functional role of *OsRDR1* in regulating endogenous gene expression in rice. By smRNA deep-sequencing, we found that extensive alteration in smRNA clusters occurred across each of the 12 rice chromosomes in the mutant, indicating a role of *OsRDR1* in smRNA biogenesis and/or titration in rice. We also found that at least some of the gene expression changes are correlated with differences in miRNAs. We further showed that changes in smRNAs can be concomitant with locus-specific alteration of cytosine methylation primarily of the CHH contexts, thus linking *OsRDR1* to DNA methylation in rice. Finally, we showed that whereas no apparent phenotypic abnormality was associated with loss of function of *OsRDR1*, ephemeral phenotypic fluctuations could be generated by various short-term abiotic stress conditions as a result of *OsRDR1* mutation, suggesting a role of *OsRDR1* in plant abiotic stress response.

## Methods

### Plant materials

Based on information about the rice retrotransposon *Tos17* insertion lines (http://tos.nias.affrc.go.jp/), we obtained a line (#RDR704) of rice cultivar Hitomebore with *Tos17* being inserted into the second exon of *OsRDR1* in a heterozygous state (accession # H0643). According to BlastN search at the NCBI website (http://blast.ncbi.nlm.nih.gov/Blast.cgi), we found that rice contains a single copy of the insert gene (*OsRDR1*). The three *OsRDR1* genotypes, WT (*RDR1/RDR1*), heterozygous (*RDR1/rdr1*) and homozygous mutant (*rdr1/rdr1*) were identified by two pairs of specific primers (Figure 1a). Specifically, WT was identified by a pair of primers anchored within the *OsRDR1* gene but flanking the *Tos17* insertion site; homozygous mutant was identified by a pair primers with on anchored to the *OsRDR1* gene and the other one targeting to the terminal of *Tos17*; and the heterozygote was identified by combinations of both types of primers. The heterozygotes of *OsRDR1*(+/−) were selfed for five successive generations, and in each of the first four generation (S1-S4) only heterozygous individuals were selected based on PCR identification. At the last generation (S5), the newly segregated homozygous mutant, i.e., *OsRDR1*−/− (designated as *Osrdr1*), and its sibling wild type (WT), i.e., *OsRDR1*+/+, were selected and propagated for an additional generation to have sufficient seeds for this study. In this way, the mutant and WT should be genetically identical except for the locus in concern, i.e., *OsRDR1*. Seeds of the two genotypes were thoroughly washed with distilled water and then germinated in the dark in Petri dishes containing distilled water at 28°C. After a 2-day incubation, germinated seeds were transferred to a greenhouse at 26°C under 16 h/8 h light/dark regime for the four kinds of abiotic stress treatments: 0.15 mMol/L NaCl (salt), 0.25 mMol/L CuSO_4_ (heavy metal Cu^2+^), 0.25 mMol/L HgCl_2_ (heavy metal Hg^2+^), 1 mMol/L Sodium nitroferricyanide(III) dehydrate (SNP, for NO stress) in Hoagland nutrient solution for 7- day. Mock controls (CK) were grown in parallel. Then, all seedling plants were transplanted to normal paddy field. Plants were surveyed at appropriate growth and developmental stages, seedling, heading and maturity.

Genomic DNA was isolated from seedlings of *Osrdr1* and WT at the same developmental stage using a modified CTAB method. Total RNA was isolated from the same seedlings with the Trizol Reagent (Invitrogen) according to the manufacturer’s instructions. The RNA was then treated with RNase-free DNase I (Invitrogen) to eliminate possible genomic DNA contamination before being reverse transcribed with the SuperScript RNase H- Reverse Transcriptase (Invitrogen).

### SmRNA library construction and sequencing

Total RNA was prepared for smRNA sequencing based on the Illumina Sample Preparation Protocol. The samples were quantified and equalized so that equivalent amounts of RNA from *Osrdr1* and WT were analyzed. In brief, total RNA was purified by electrophoretic separation on a 15% TBE-urea denaturing PAGE gel and smRNA regions corresponding to the 15–30 nucleotide bands in the marker lane were excised and recovered. The 15–30 nt smRNAs were 5’ and 3’ RNA adapter-ligated by T4 RNA ligase and at each step length validated and purified by urea PAGE gel electrophoretic separation. The adapter-ligated smRNA was subsequently transcribed into cDNA by Super- Script II reverse transcriptase (Invitrogen) and PCR amplified, using primers that anneal to the ends of the adapters. The amplified cDNA, too, was purified and recovered. The final quality of the library was ensured by validation of the size, purity and concentration using an Agilent Technologies 2100 Bioanalyzer. The two constructed cDNA libraries subsequently underwent Solexa/Illumina’s proprietary flowcell cluster generation and bridge amplification.

### Analysis of smRNA clusters

SmRNA reads of 18-26 nt in size were counted within every sliding 100 bp window along the rice genome. The reads were normalized to RPM (reads per million), and comparison was then made between *Osrdr1* and WT plants using the median RPM values, which are denoted as X for *Osrdr1* and Y for WT plants. The fold value was calculated by the formula log_2_X-log_2_Y = log_2_(X/Y).

### Affymetrix GeneChip® Rice Genome Array

The microarray transcriptional profiling was performed by the Affymetrix, Inc. in the Gene Company Ltd. (Shanghai, China), using procedures described in the GeneChip® Expression Analysis Technical Manual.

### Real-time quantitative (q) reverse transcriptase (RT)-PCR analysis

The qRT-PCR experiments were performed using SYBR Premix *Ex* Taq (TOYOBO) according to the manufacturer’s instruction on a Roche LightCycler480 apparatus (Roche Inc.). The primers for amplifying the 18 studied genes were designed using the Primer 5 software and listed in Additional file 6. Primers for qRT-PCR analysis of transposase genes were described in a previous report [40]. Expression of a rice *β-actin* gene, *eEF* gene and *UBQ5* gene were used as internal control with the primer pairs of5’-atgccattctccgtctt and 5’-gctcctgctcgtagtc; 5’-tttcactcttggtgtgaagcagat and 5’-gacttccttcacgatttcatcgtaa; 5’-accacttcgaccgccactact and 5’-acgcctaagcctgctggtt, respectively. Conditions of RT-qPCR were as reported [40].

### Hemi-nested RT-PCR for detecting miRNA expression

Four miRNA-specific oligonucleotide primers were designed according to a previous report [41,42] to reverse transcribe the specific miRNAs, and four pairs of specific primers corresponding to the RT primers were designed to amplify the cDNA (Additional file 6). RT-PCR was performed as previously described [41,42].

### Bisulphite sequencing

DNA (~1 μg) from each plant was treated with bisulphite using the EZ DNA Methylation-Gold Kit and amplified using specific primers (Additional file 6). Bisulphite PCR product was cloned into vector NTI and positive clones were sent for sequencing. The results were analyzed using analysis software on the website http://katahdin.mssm.edu/kismeth based on a previous report [43].

## Competing interests

The authors declare that they have no competing interests.

## Authors’ contributions

NW and DZ carried out major parts of the experiments, analyzed the data and drafted the manuscript. ZHW, HWX, JM, HW, WH, YL, XYL, CYZ, OXF and NL participated in all the experiments. BL and MBW designed the work and finalized the manuscript. All authors read and approved the final manuscript.

## Supplementary Material

Additional file 1: Figure S1 Chromosomal distribution of differential smRNA clusters between *Osrdr1* and WT for a selected subset smRNAs in the size ranges of 20–24 nt. Where x axis is the length of chromosome (Per 100 bp window) and y axis is the value of different RPMs (log value, base 2). The vertical blue lines denote centromeric regions in each chromosome. Click here for file

Additional file 2: Table S1 List of expression of known miRNAs between *Osrdr1 and* wide-type. Click here for file

Additional file 3: Figure S2 Putative novel miRNAs identified from *Osrdr1* and wide-type. (a) The sequences, expression status including read counts, and genomic locations of the novel miRNAs. 5p, the mature miRNA sequence resides in 5’ half of the predicted stem-loop structure; 3p, the mature miRNA sequence resides in 3’ half of the predicted stem-loop structure. mfe, minimum free energy. (b) The predicted stem-loop structure of precursor RNA of the novel miRNAs. The mature miRNA sequence inside the stem-loop is indicated by a red line, and the 5’ to 3’ direction of a miRNA is indicated by an arrowhead. Click here for file

Additional file 4: Figure S3 (a) Correlation of expression of known miRNAs and their targets. (b) Pairwise comparison between expression levels of known miRNAs and their targets. Yellow and blue columns represent target and miRNA expression levels, respectively. *Y*-axis indicates the values of log_2_ fold change. Click here for file

Additional file 5: Figure S4 Regional association between smRNA clusters and DNA methylation for each of the 10 assayed genomic loci from two transposable elements (TEs), *Tos17* (four regions) and *Pong* (three regions) and three genes (one region each). The red, blue and green circles denote for CG, CHG and CHH sequence contexts, respectively, wherein the filled ones are methylated and empty ones are unmethylated. Click here for file

Additional file 6: Table S2 Primers for qRT-PCR assay of 18 genes, bisulfite sequencing of 10 loci and semi-nested qRT-PCR analysis of four miRNAs. Click here for file
