# Supplementary figures and images for: Mutation of the RDR1 gene caused genome-wide changes in gene expression, regional variation in small RNA clusters and localized alteration in DNA methylation in rice
Source: BMC Plant Biol. 2014 Jun 30;14:177. doi: 10.1186/1471-2229-14-177 (PMC4083042; doi:10.1186/1471-2229-14-177)

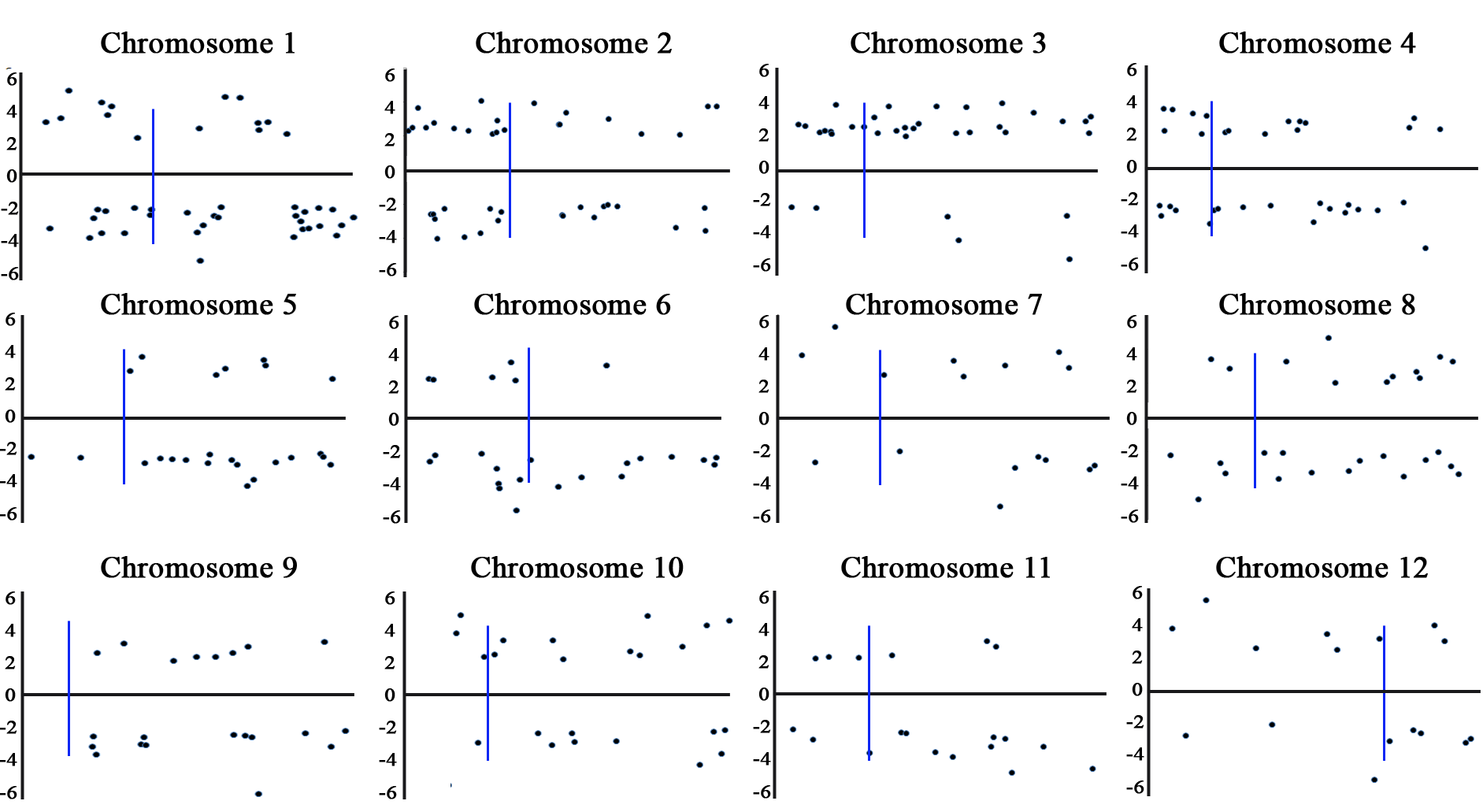

Supplement: Additional file 1: Figure S1 — Chromosomal distribution of differential smRNA clusters between Osrdr1 and WT for a selected subset smRNAs in the size ranges of 20–24 nt. Where x axis is the length of chromosome (Per 100 bp window) and y axis is the value of different RPMs (log value, base 2). The vertical blue lines denote centromeric regions in each chromosome. [file 1471-2229-14-177-S1.tiff]

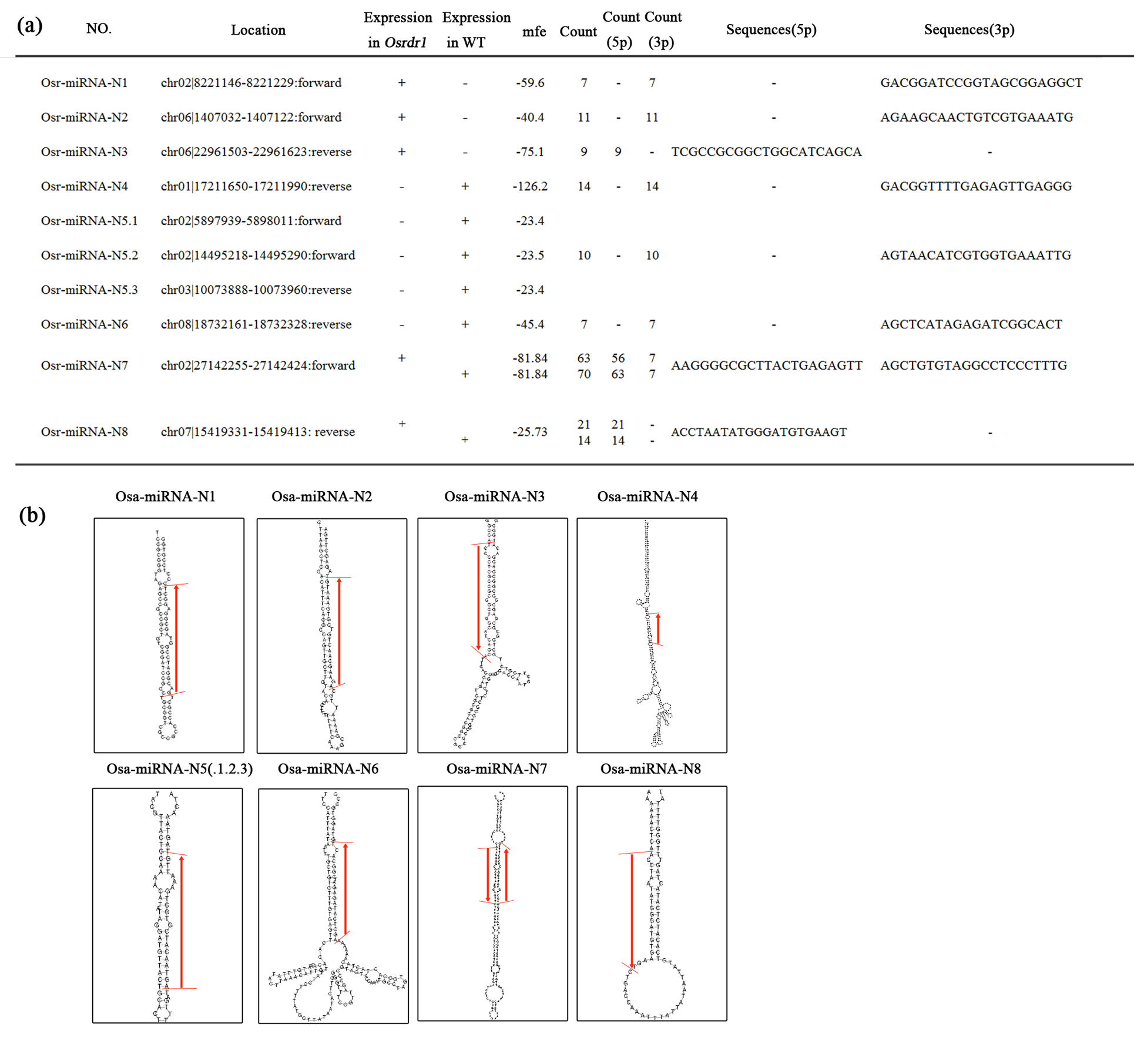

Supplement: Additional file 3: Figure S2 — Putative novel miRNAs identified from Osrdr1 and wide-type. (a) The sequences, expression status including read counts, and genomic locations of the novel miRNAs. 5p, the mature miRNA sequence resides in 5’ half of the predicted stem-loop structure; 3p, the mature miRNA sequence resides in 3’ half of the predicted stem-loop structure. mfe, minimum free energy. (b) The predicted stem-loop structure of precursor RNA of the novel miRNAs. The mature miRNA sequence inside the stem-loop is indicated by a red line, and the 5’ to 3’ direction of a miRNA is indicated by an arrowhead. [file 1471-2229-14-177-S3.tiff]

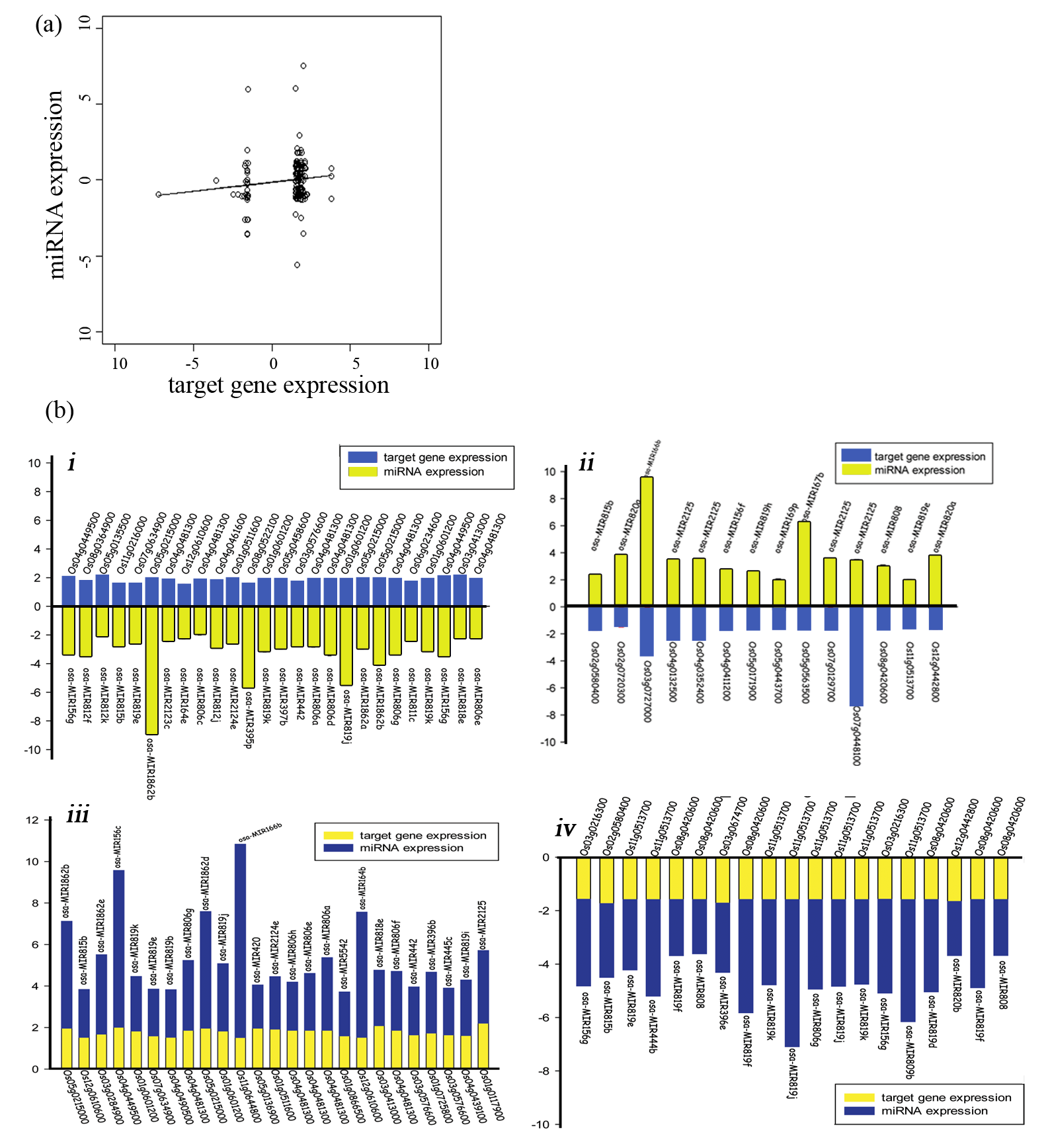

Supplement: Additional file 4: Figure S3 — (a) Correlation of expression of known miRNAs and their targets. (b) Pairwise comparison between expression levels of known miRNAs and their targets. Yellow and blue columns represent target and miRNA expression levels, respectively. Y-axis indicates the values of log2 fold change. [file 1471-2229-14-177-S4.tiff]

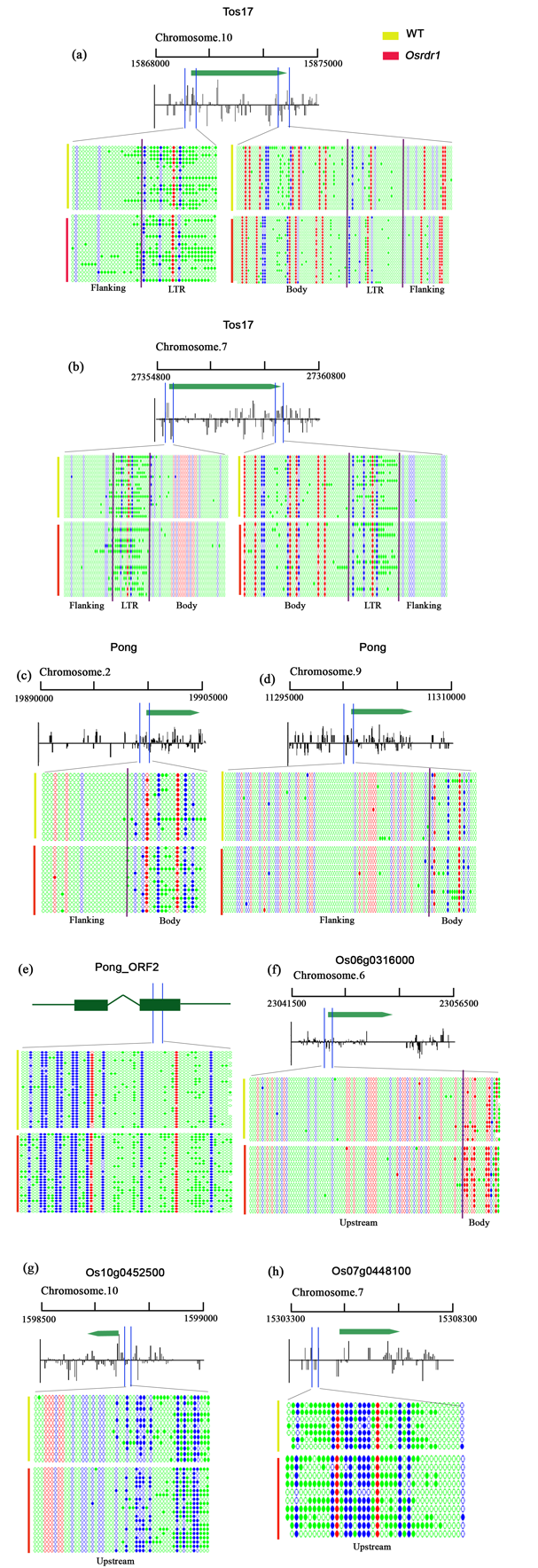

Supplement: Additional file 5: Figure S4 — Regional association between smRNA clusters and DNA methylation for each of the 10 assayed genomic loci from two transposable elements (TEs), Tos17 (four regions) and Pong (three regions) and three genes (one region each). The red, blue and green circles denote for CG, CHG and CHH sequence contexts, respectively, wherein the filled ones are methylated and empty ones are unmethylated. [file 1471-2229-14-177-S5.tiff]
